# Microstructural MRI Correlates of Cognitive Impairment in Multiple Sclerosis: The Role of Deep Gray Matter

**DOI:** 10.3390/diagnostics11061103

**Published:** 2021-06-16

**Authors:** Marco Pitteri, Ilaria Boscolo Galazzo, Lorenza Brusini, Federica Cruciani, Caterina Dapor, Damiano Marastoni, Gloria Menegaz, Massimiliano Calabrese

**Affiliations:** 1 Neurology Section, Department of Neurosciences, Biomedicine and Movement Sciences, University of Verona, 37134 Verona, Italy; caterina.dapor@univr.it (C.D.); damiano.marastoni@univr.it (D.M.); 2 Department of Computer Science, University of Verona, 37134 Verona, Italy; ilaria.boscologalazzo@univr.it (I.B.G.); lorenza.brusini@univr.it (L.B.); federica.cruciani@univr.it (F.C.); gloria.menegaz@univr.it (G.M.)

**Keywords:** multiple sclerosis, cognitive impairment, diffusion MRI, DTI, 3D-SHORE

## Abstract

Although cognitive impairment (CI) is frequently observed in people with multiple sclerosis (pwMS), its pathogenesis is still controversial. Conflicting results emerged concerning the role of microstructural gray matter (GM) damage especially when involving the deep GM structures. In this study, we aimed at evaluating whether differences in cortical and deep GM structures between apparently cognitively normal (ACN) and CI pwMS (36 subjects in total) are present, using an extensive set of diffusion MRI (dMRI) indices and conventional morphometry measures. The results revealed increased anisotropy and restriction over several deep GM structures in CI compared with ACN pwMS, while no changes in volume were present in the same areas. Conversely, reduced anisotropy/restriction values were detected in cortical regions, mostly the pericalcarine cortex and precuneus, combined with reduced thickness of the superior frontal gyrus and insula. Most of the dMRI metrics but none of the morphometric indices correlated with the Symbol Digit Modality Test. These results suggest that deep GM microstructural damage can be a strong anatomical substrate of CI in pwMS and might allow identifying pwMS at higher risk of developing CI.

## 1. Introduction

Multiple sclerosis (MS) is a chronic, inflammatory, neurodegenerative disease of the central nervous system (CNS), characterized by the accumulation of white matter (WM) and gray matter (GM) damage [1,2]. One of the most frequent and early clinical manifestation of MS is cognitive impairment (CI) [3], which reflects a degree of neuro-axonal loss and/or dysfunction [3,4,5].

Different neural substrates are likely to contribute to global CI and impairment of specific cognitive domains in people with MS (pwMS). PwMS with more severe CI have been shown to be characterized by greater cortical atrophy [6], greater deep GM atrophy [7], and higher number of cortical lesions [8,9], as well as lesion location in strategic WM regions, WM microstructural damage, and abnormal patterns of cerebral activation [10,11].

Nonetheless, the strength of the association between conventional neuroimaging findings and the cognitive manifestations of MS disease remains modest. This is likely due to the lack of specificity of conventional Magnetic Resonance Imaging (MRI) sequences for evaluating the heterogeneous pathological substrates of MS, to their inability to provide accurate estimates of damage outside focal lesions, and to the fact that they cannot inform on the functional and structural reorganization of the CNS after tissue damage [12].

Diffusion MRI (dMRI) sequences marked a step toward a more complete characterization of the cortical/subcortical tissue modulations beneath the CI in pwMS, going beyond the information provided by traditional morphometric measures. This technique allows to non-invasively quantify the microstructural properties of brain tissues in vivo, by relying on ad-hoc dMRI acquisitions and analytical/compartmental models for deriving different dMRI indices. The well-known diffusion tensor imaging (DTI) [13] has shown good sensitivity in detecting brain damage in pwMS, revealing diffusivity and anisotropy abnormalities in focal lesions and normal-appearing WM [14] also related to CI [15]. When considering GM tissues, while previous DTI studies consistently showed increased mean diffusivity (MD), diverging findings have emerged for fractional anisotropy (FA) so far, with increased [16], decreased [17] or no change [16,17,18] in the cortical and subcortical normal-appearing GM of pwMS.

In the last few years, the availability of more sophisticated dMRI models and acquisition sequences have opened the way for more systematic studies on pwMS with different disease phenotypes and for overcoming the technical limitations inherent to simple methods as DTI. In a recent study, Brusini and colleagues [19] focused on a new advanced dMRI models, based on an analytical formulation that relies on the estimation of the Ensemble Average Propagator (EAP) by using the Simple Harmonic Oscillator-based Reconstruction and Estimation (SHORE) model. The results showed that dMRI indices, especially those derived from 3D-SHORE, allowed capturing microstructural alterations between RRMS and primary progressive MS (PPMS) phenotypes highlighting the prominence of dMRI in decrypting microstructural differences between these MS phenotypes.

Only few studies have investigated this technique in patients with and without CI, almost exclusively focusing on WM damage rather than cortical and subcortical GM [20,21,22,23]. Moreover, the criteria adopted to classify these patients are also variegated in the current literature, precluding a straightforward generalization of the results.

In the present study, we aimed at investigating whether advanced dMRI techniques, in particular 3D-SHORE derived measurements, might allow to detect GM differences in cortical and deep brain structures between apparently cognitively normal (ACN; [24]) and CI pwMS compared with DTI and conventional morphometry measures.

## 2. Materials and Methods

### 2.1. Study Population

Thirty-six patients having MS according to the recent MS diagnostic criteria [25] were evaluated at the MS Centre of Verona University Hospital (Verona, Italy) and were enrolled in the present study.

Each patient underwent a neurological examination, including EDSS assessment (Expanded Disability Status Scale [26]), a 3T MRI scan, and a battery of neuropsychological tests.

At the time of neuropsychological testing, 15 patients were not treated with specific disease-modifying therapy for MS, whereas 9 were treated with dimethyl-fumarate, 7 with fingolimod, 2 with ocrelizumab, 2 with teriflunomide, and 1 with azathioprine.

Among the whole MS cohort, neuropsychological assessment classified 11 (30%) pwMS as being apparently cognitive normal (ACN; 0 tests below the cut-off) and 25 (70%) pwMS as having CI (1 or more test below the cut-off; see also [27,28]). Demographic and clinical characteristics of the MS cohort and each group of pwMS are listed in Table 1.

The study was approved by the local Ethics Committee and written informed consent was collected from all participants (MSBioB Biological bank—A.O.U.I. Verona, protocol number 66418, 25 November 2019).

### 2.2. Neuropsychological Assessment

MS patients were tested with a battery of neuropsychological tests, comprising of the Brief Repeatable Battery of neuropsychological tests (BRB-NT; [29] and the Stroop Test (ST; [30]). The total number of tests considered was 11 (nine subtests of the BRB-NT, two subtests of the ST). The BRB-NT is composed of tests of verbal learning and delayed memory recall (Selective Reminding Test, SRT), visuospatial learning and delayed memory recall (10/36 Spatial Recall Test, SPART), visual information processing speed and attention (Symbol Digit Modalities Test, SDMT), auditory information processing speed, attention, and calculation (Paced Auditory Serial Addition Task, PASAT), and semantic verbal fluency with double category (Word List Generation, WLG). The ST is a test of attention and automatic response inhibition with time (ST-EIT) and errors (ST-EIE) as dependent variables. Test performance was considered impaired based on cut-off scores (5th percentile) derived from the Italian normative data [29].

### 2.3. MRI Data Collection

All subjects underwent an MRI acquisition on a 3T Philips Achieva scanner (Philips Medical Systems, Best, The Netherlands) equipped with an 8-channel head receiver coil. The protocol included three volumetric structural sequences, namely T1-weighted Fast Field Echo ([T1w], repetition time [TR]/echo time [TE] = 8.1/3 ms, flip angle [FA] = 8°, field of view [FOV] = 240 × 240 mm^2^, 1-mm isotropic resolution, 180 slices), Turbo Spin Echo T2-weighted ([T2w], TR/TE = 2500/228 ms, FA = 90°, FOV = 256 × 256 mm^2^, 1-mm isotropic resolution, 180 slices), and Fluid-Attenuated Inversion Recovery ([FLAIR], TR/TE = 8000/290 ms, TI = 2356 ms, FA = 90°, FOV = 256 x 256 mm^2^, 0.9 × 0.9 × 0.5 mm^3^ resolution, 180 slices), followed by a two-shells dMRI acquisition (TR/TE = 9300/109 ms, FA = 90°, FOV = 112 × 112 mm^2^, 2-mm isotropic resolution, 62 slices, b-values = 700/2000 s/mm^2^ with 32/64 gradient directions and 7 b0 volumes).

### 2.4. MRI Data Processing and Features Extraction

The Tortoise DIFFPREP pipeline (https://tortoise.nibib.nih.gov/tortoise, accessed on 15 May 2020) was used for re-sampling the dMRI data, followed by corrections for motion, eddy-current, and EPI distortions. Subsequently, brain extraction and masking were performed employing FSL v6.0 software (FMRIB, Oxford, UK; https://fsl.fmrib.ox.ac.uk/fsl/fslwiki/, accessed on 15 May 2020). For each patient, the transformation matrix of the rigid registration of the T1w image to the mean b0 volume was estimated. Finally, the DTI and the 3D-SHORE models were fitted to the preprocessed dMRI signal data using DIPY (https://dipy.org/, accessed on 30 June 2020).

Concerning the standard structural images processing, a first step of lesion detection was performed for each subject involved in the study. The rigid registration of each FLAIR image to the T1w was performed in FSL. The Lesion Prediction Algorithm (LPA) in the Lesion Segmentation Toolbox (LST) for SPM12 (www.statistical-modelling.de/lst.html, accessed on 29 May 2020) was then applied to the co-registered FLAIR image to automatically segment the lesions, which were subsequently filled with the corresponding algorithm available in LST. All T1w filled images were used as input for the FreeSurfer software (Harvard University, Boston, MA, USA, http://surfer.nmr.mgh.harvard.edu/, accessed on 7 July 2020) to parcellate each individual brain into 112 anatomical regions of interests (ROIs). Referring to the recent literature that showed the most common involved GM structures related to MS [2,31,32], a subset of the resulting ROIs were selected for the subsequent analyses: thalamus (Thal), caudate (Cau), putamen (Put), hippocampus (Hipp), insula (Ins), precuneus (Pre), superior-frontal gyrus (SFG), posterior cingulate cortex (PCC), lateral occipital cortex (LOC), lingual gyrus (LgG), and pericalcarine cortex (PC). The feature extraction started with the derivation of several microstructural indices from the EAP calculated, fitting the models. In particular, the EAP, which is the probability that the water molecules travel a displacement *x* within the acquisition diffusion time, allows the computation of Fractional Anisotropy (FA) and Mean Diffusivity (MD) [13] from the DTI model fitting, and of Generalized Fractional Anisotropy (GFA), Propagator Anisotropy (PA), Mean Square Displacement (MSD), Return To the Origin/Axes/Plane Probability (RTOP, RTAP, and RTPP, respectively) from the 3D-SHORE model [33,34,35,36]. Among these indices, FA, GFA, and PA bring information about the local anisotropy of the diffusion process and take higher values when one main diffusion direction is present. Accordingly, they are typically higher in WM, in which diffusion is restricted by the myelin sheaths, particularly in tracts with uniform fiber alignment, whereas diffusion in GM is less bounded and more isotropic. Conversely, MD and MSD express the displacement of the spins in unit time and are higher for unconstrained diffusion. Finally, RTPP, RTAP, and RTOP, which are related to the geometry of the pore constraining the diffusion process, take on higher values when diffusion is highly restricted in one, two, or three spatial dimensions, respectively.

Once all the dMRI indices were estimated, the previously calculated transformation matrix from the T1w to the dMRI space was individually applied to each parcellation, and the selected ROI masks were used to calculate the regional dMRI indices’ values. The median value for each ROI and participant was finally derived.

In addition, volume and thickness measurements were extracted from the FreeSurfer statistics for each subcortical and cortical ROI, respectively, and were used as main morphometric descriptors. The average values across the two hemispheres were obtained, and the normalization by the estimated total intracranial volume (eTIV) was performed for volume measures only.

### 2.5. Statistical Analysis

Differences between the groups on the neuropsychological test scores were assessed with one-way analysis of covariance (ANCOVA) followed by pairwise comparisons with post-hoc tests, where appropriate. Age, gender, and disease duration were included as covariates in line with previous studies [37]. Conversely, group-differences between the extracted MRI features were evaluated by performing a two-way ANCOVA, separately for each microstructural index and morphometric measure. In particular, the group (GROUP) and the brain region (ROI) were used as fixed factors, while the three covariates were added to the model as before. Post-hoc tests adjusted for multiple comparisons with Bonferroni corrections were computed for the significant interactions. For all statistical tests, the significance threshold was set at *p* < 0.05.

Non-parametric analyses were finally performed to assess the associations between MRI measures and each neuropsychological test, separately for all the considered ROIs. To this end, the Spearman correlation was calculated between each individual MRI feature and neuropsychological score, and the results were adjusted for multiple comparisons (false discovery rate [FDR]).

## 3. Results

### 3.1. Neuropsychological Performance between ACN and CI Patients

Age distribution and EDSS were different between the two groups, as revealed by the unpaired two-sample t-test (*p* < 0.05). Conversely, no differences were present in terms of education, gender, and disease duration as reported in Table 1 (*p* > 0.05).

The ANCOVA results showed that CI pwMS performed worse in almost all neuropsychological tests, except the PASAT-2 and the Stroop test. A detailed description of the ANCOVA results is provided in Supplementary Materials.

### 3.2. Qualitative Analysis of Microstructural Indices

**Figure 1:**
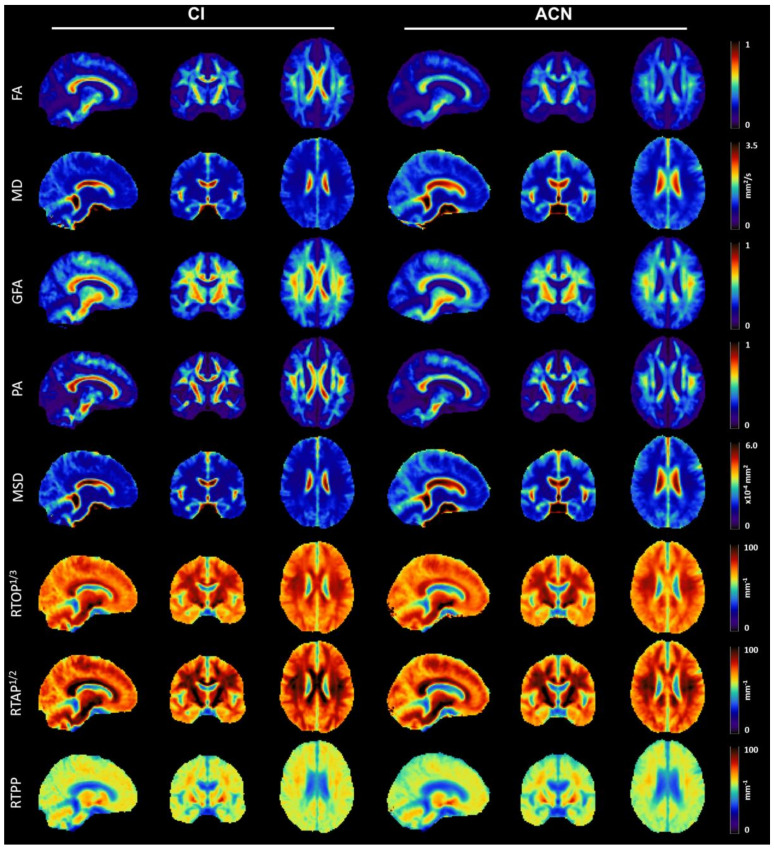
Microstructural maps of values averaged across cognitive impaired (CI) and apparently cognitive normal (ACN) patients, respectively. The representative sections reported here are in MNI space and are displayed in radiological convention. FA = fractional anisotropy; MD = mean diffusivity; GFA = generalized fractional anisotropy; PA = propagator anisotropy; MSD = mean square displacement; RTOP = return to the origin probability; RTAP = return to the axis probability; RTPP = return to the plane probability.

The average parameter maps calculated for each dMRI index over the two groups are reported in Figure 1. For ease of comparison across restriction indices, the cubic- and square-root of RTOP and RTAP were calculated and displayed, both expressed in mm^−1^. As expected, MD and MSD reached the highest values in areas of unrestricted diffusion, in particular the cerebrospinal fluid, with a reverse pattern compared with all the other six indices. High anisotropy values can be observed for FA, GFA, and PA in regions where diffusion mostly happens along one preferred direction, such as the corpus callosum. RTOP, RTAP, and RTPP have similar contrast though there are slight differences in appearance due to the difference in the restriction dimension that is respectively probed. In particular, RTPP shows the lowest contrast across tissues being sensitive to the compartment mean apparent length, whereas RTAP had clearly higher values in regions such as the corpus callosum due to its sensitivity to the compartment mean apparent cross-sectional area. RTOP being sensitive to the compartment mean apparent volume, it showed a contrast similar to RTAP, though slightly less marked in the contrast between WM and GM.

While qualitative differences between CI and ACN subjects can be appreciated in WM areas, especially in terms of anisotropy, no evident inter-group differences can be detected over GM tissues by visual inspection.

### 3.3. Statistical Analysis of MRI-Derived Numerical Biomarkers

ANCOVA analyses performed on GM regional values identified a statistically significant two-way interaction (GROUP*ROI) for all the anisotropy indices (FA: F(10,371) = 3.07, *p* = 0.001, $\eta_{p}^{2}$ = 0.077; GFA: F(10,371) = 2.61, *p* = 0.005, $\eta_{p}^{2}$ = 0.066; PA: F(10,371) = 3.00, *p* = 0.001, $\eta_{p}^{2}$ = 0.075), RTAPand RTOP (F(10,371) = 2.24, *p* = 0.015, $\eta_{p}^{2}$ = 0.057 and F(10,371) = 2.20, *p* = 0.017, $\eta_{p}^{2}$ = 0.056, respectively). The results of all the significant post-hoc Bonferroni tests are summarized in Table 2. Concerning the three anisotropy indices, post-hoc tests revealed significant between-group differences in subcortical areas, more precisely the Cau (FA, GFA, PA), Put (FA, GFA), and Thal (PA), with increased values in CI compared with ACN (*p*_Bonf_ < 0.05). RTAP and RTOP showed similar post-hoc significant differences, with increased restriction values in the putamen when comparing CI vs ACN. In addition, in this case, several common cortical regions emerged as significantly different, namely the PCC, LgG, PC, and Pre, with all of them featuring reduced values in the CI group. Of note, the last two regions also showed a significantly reduced anisotropy in CI subjects as measured by GFA (*p*_Bonf_ < 0.05). In most of the cases, the effect sizes as expressed by the Hedge’s *g* factors were large (>|0.8|), especially for FA and PA in deep GM structures.

In terms of atrophy, ANCOVA analyses on regional morphometry identified a statistically significant two-way interaction (GROUP*ROI) for the thickness measures (F(6,235) = 3.46, *p* = 0.003, $\eta_{p}^{2}$ = 0.081), with post-hoc Bonferroni tests showing reduced values in CI compared with ACN for SFG (−0.117 ± 0.04, *p*_Bonf_ = 0.004, Hedge’s *g* = −1.012) and Ins (−0.16 ± 0.04, *p*_Bonf_ = 0.0001, Hedge’s *g* = −1.359). Conversely, the two-way interaction did not reach the statistical significance for the volume measures.

### 3.4. Associations between MRI-Derived Numerical Biomarkers and Neuropsychological Tests

**Figure 2:**
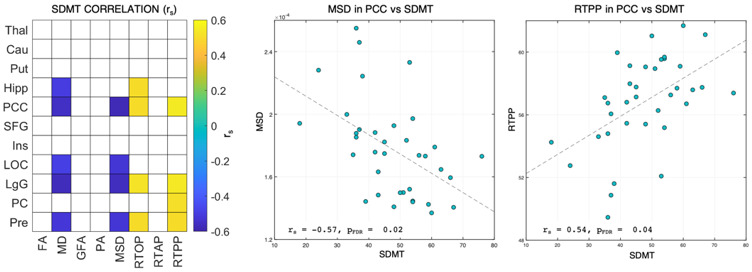
Spearman correlations (r_S_ values, *p*_FDR_ < 0.05) between each regional microstructural index value and SDMT scores (left). Significant associations are color-coded, while white squares indicate no significant associations. Scatter plots representing the associations between SDMT scores with MSD (center) and RTPP (right) values in the PCCare also reported. **Legend.** SDMT = Symbol Digit Modalities Test; Thal = thalamus; Cau = caudate; Put = putamen; Hipp = hippocampus; PCC = posterior cingular cortex; SFG = superior frontal gyrus; Ins = insula; LOC = lateral occipital cortex; LgG = lingual gyrus; PC = pericalcarine cortex; Pre = precuneus; FA = fractional anisotropy; MD = mean diffusivity; GFA = generalized fractional anisotropy; PA = propagator anisotropy; MSD = mean square displacement; RTOP = return to the origin probability; RTAP = return to the axis probability; RTPP = return to the plane probability.

Among all the neuropsychological tests, the SDMT showed several significant correlations with the diffusivity indices (MD and MSD), RTOP, and RTPP (*p*_FDR_ < 0.05), while associations with the anisotropy measures and RTAP did not reach the statistical significance (Figure 2). In terms of brain regions, the strongest associations were found for PCC and LgG in all these dMRI indices (r_s_ > |0.5|, *p*_FDR_ < 0.05), though with opposite signs. More specifically, a negative association was visible for the diffusivity measures, with MSD reaching the highest values, while the restriction indices were positively associated in these regions. Significant correlations were also found in Pre for the four indices, again with the opposite trend (r_s_ = −0.52, *p*_FDR_ < 0.05 for MD and MSD, r_s_ = 0.5, *p*_FDR_ < 0.05 for RTOP and RTPP), while MD and RTOP revealed significant associations in Hipp (r_s_ = −0.49 and r_s_ = 0.50, *p*_FDR_ < 0.05, respectively).

For all the other neuropsychological tests, few correlations emerged as significant, in particular between WLG and MD in Pre (r_s_ = −0.50, *p*_FDR_ < 0.05), and between the SRT-D and RTOP in Put (r_s_ = −0.51, *p*_FDR_ < 0.05).

Regarding the morphometric measures, only the normalized volume of Put showed a significant correlation with one of the neuropsychological measures (ST-EIE, r_s_ = −0.52, *p*_FDR_ < 0.05).

## 4. Discussion

### 4.1. ACN and CI Groups Differed in Neuropsychological Performance

The present results showed that pwMS with CI performed worse as compared with ACN group in all administered tests. This is in line with a previous study in which all cognitive scores were significantly lower in pwMS with CI compared with non-CI patients [38]. Although the definitions and the diagnostic criteria of CI differ between studies [39], the present results confirm the clinical usefulness of this conservative approach to effectively classify patients with CI [27,28].

### 4.2. Between-Group Changes in Deep GM Are Depicted by dMRI Measures Only

The ACN and CI groups differed in several dMRI measures, specifically in the anisotropy indices (FA, GFA, PA) as well as restriction indices (RTAP and RTOP). All these indices identified alterations in subcortical structures such as putamen and caudate, with increased values in CI compared with ACN pwMS. Conversely, no deep GM volume differences were detected between the two groups possibly suggesting a similar level of atrophy in these areas. So far, only few studies have investigated the microstructural modulations in pwMS with and without CI and usually only DTI-derived indices have been assessed, generally restricting the analyses to WM tracts. In particular, Preziosa et al. (2016) reported several modifications in patients with CI compared with those cognitively preserved (CP), including increased MD for major WM tracts and only limited FA alterations [20]. Similarly, Daams et al. (2016) focused on analogous patients’ groups and demonstrated severe microstructural variations in different WM tracts, such as reduced FA and increased MD over the corpus callosum and anterior thalamic radiations [22].

High anisotropy and high restriction are typical aspects of WM [13,33,34]. However, the focus of our study was on GM where the microstructural indices interpretation is even more difficult due to the great complexity of this tissue in which many different cellular structures coexist. Nevertheless, it has been evidenced that the dMRI signal in the cortex appears to be layer-dependent, and even the simplest dMRI model (i.e., DTI) can inform on GM microstructure [40]. Moreover, the fiber orientation density seems to be sensitive to this specific variation, as suggested by ex vivo studies (see [41] for a review on the topic). In this context, the higher values of these metrics (i.e., FA, GFA, PA, RTOP, and RTAP in our study) in CI compared with ACN may indicate a lower orientation dispersion of cellular structures in deep GM.

The altered microstructural patterns we detected in the deep GM in terms of anisotropy and restriction provide novel information about localized changes in patients with CI and confirmed that deep GM structures are not spared in MS [42] and are highly associated with cognitive decline [31,43]. Indeed, since the decrease in information-processing speed (IPS) is one of the main features of cognitive impairment in MS [44], damage to these deep GM structures and their WM connections has been linked to CI [32,45,46,47,48]. All these findings highlight the importance of detecting early microstructure abnormalities when considering the effect of MS pathology on network performance, since deep GM, combined with cortical GM, have a pivotal role as hub in several brain networks associated with cognitive functioning [49]. Moreover, they push toward the investigation of possible microstructural alterations in the fiber bundles connecting these regions, to provide a complete assessment of the link between dMRI damage and CI.

### 4.3. Cortical Thickness and Microstructure Are Selectively Different between the Two Groups in Cortical Areas

Decreased anisotropy (GFA) and restriction (RTAP, RTOP) were found in several cortical areas for CI pwMS, particularly in the pericalcarine and the precuneus cortex. The lower values of these indices in CI patients suggest a disruption of the microstructure integrity [13,34] that may contribute to the cognitive decline. Conversely, classical DTI measures did not depict any variation involving cortical structures.

To the best of our knowledge, only a previous study investigated whether microstructural differences between CI and CP subjects are present in cortical GM areas, though relying only on the two main DTI-based indices and focusing on the entire skeletonized cortex after removal of the cerebellum and deep GM [21]. The authors reported increased MD values in CI compared with CP, while the opposite pattern was shown by FA. This pattern of decreased anisotropy in specific cortical areas was also demonstrated in our study by more advanced dMRI indices (GFA), albeit being not associated with increased diffusivity.

In terms of atrophy, we found significant cortical thinning in CI compared with ACN in the superior frontal gyrus and insula. The lack of other differences between CI and ACN may be due to the low number of patients included in the study. Nevertheless, our results are in line with previous studies showing that the damage of the superior frontal cortex seems to play a crucial role in CI in pwMS [50] and being linked to verbal learning and delayed memory recall abilities [51]. The insula, especially the anterior part, has a central role in integrating different functional systems involved in affective and cognitive processing [52,53]. Reduced neocortical volume and a widespread pattern of regional GM atrophy in the cortex have been frequently associated with CI in pwMS [54].

Our findings suggested a close relationship between CI and brain modulations in regions that are well-known as “network hubs”, such as the pericalcarine cortex, the precuneus and the posterior cingulate cortex, and are particularly sensitive to alterations and pathology in the brain [55]. Among them, the posterior cingulate cortex, a highly connected brain region associated with cognitive functioning, has been shown to be a primary area affected in MS disease. Altered posterior cingulate cortex functioning was reported in pwMS with and without CI, further supporting the hypothesis that abnormalities in this area could serve as a marker of CI, independently from atrophy [56].

These results might reflect the altered pathological process occurring in MS since the early stages of the disease, such as myelin and neuronal loss, leaving extracellular spaces filled by water, but also by changing of the morphology and orientation of microglia and astrocyte processes due to their early activation [2,57].

### 4.4. dMRI Measures Correlate with the SDMT

The imaging results were corroborated by the correlation between the GM structures and the neuropsychological tests. SDMT was almost the unique neuropsychological test that correlates with dMRI metrics, but not with classic morphometric metrics (volumes/cortical thickness). These correlations values are in line with the results reported in a previous study [58] where a partial least squares regression emphasized a direct correlation between SDMT and RTOP/RTPP, and an indirect correlation between SDMT and MD/MSD. The SDMT is the most common test used in MS and it has been shown to be the most reliable and sensitive cognitive measure for use in MS [59]. Deficits in IPS, usually assessed with the SDMT, are among the first cognitive symptoms in pwMS [4,60]. The search for neural correlates of IPS deficits resulted in several structural and functional brain measures, including WM and GM damage, but also changes in activation and functional connectivity (FC) [61]. IPS deficits in MS have typically been explained as a consequence of WM damage, but also of cortical and deep GM atrophy [32,45,46,47,48]. This is not surprising given that cortical and deep GM regions have been related to the Default Mode Network (DMN; [62]), in which hub structures such as the medial superior frontal gyrus, posterior cingulate cortex, angular gyrus, precuneus, and the middle-, superior-, and inferior-frontal gyrus have been related to cognitive impairment, especially IPS [63,64]. A recent study by Louapre et al. (2014) showed that the volume of the posterior cingulate cortex was able to predict the strength of the functional correlation within the DMN of pwMS with CI, suggesting that disconnection in the DMN may deprive the brain compensatory mechanisms that are involved to contrast the widespread structural damage related to cognitive functioning in pwMS [65].

Moreover, DMN has been shown to be associated with IPS and SDMT performance in previous studies investigating the functional and structural connectivity patterns of this critical network [66,67]. Our findings further support this concept, suggesting that structural alterations over the DMN, as well as deep GM, affect cognitively relevant network functioning [68].

Notably, the damage to the structural brain architecture has larger consequences for IPS than functional brain changes [69]. PwMS with different severities of functional and structural damage reflected stepwise worsening of IPS: pwMS with predominant structural damage had worse IPS than pwMS with low-structural and low-functional damage and also than pwMS with low-functional damage only, suggesting that the severity of functional network changes seem to have an additive effect over structural damage on IPS performance, as a similar degree of structural damage can be accompanied with either mild or severe functional network changes, resulting in different levels of IPS [69]. The absence of a strict one-to-one relationship between the level of structural and functional damage emphasizes the value of integrating both measures; future studies should investigate this issue.

Adding information about the severity of functional changes is needed to distinguish between different levels of IPS performance in patients with similar degrees of structural damage [69]: our results suggest that dMRI indices may be reliable and add further information in detecting microstructure damage of deep GM structures that are involved in functional networks subserving IPS in pwMS.

### 4.5. Study Limitations

This study is not without limitations. First, in this cohort of patients no measures of subjective fatigue and emotional status were available; then, we cannot rule out that other factors, such as depression, anxiety, and fatigue, might have partially influenced our results. Second, the sample size of this MS cohort was relatively small: future studies should corroborate the present results in a higher number of patients. Third, no functional MRI measures have been used, although the multimodal combination of these data could help to further clarify the role of MRI-derived biomarkers for characterizing CI in pwMS.

## 5. Conclusions

To conclude, our findings revealed that different deep GM microstructure damage, detected by dMRI indices, can be the main anatomical substrate related to alterations in cognitive functioning in pwMS. The potential of dMRI indices as imaging biomarkers was also highlighted by the significant associations with the SDMT. Early microstructural alterations in critical deep GM regions might allow to identifying patients at higher risk of developing cognitive dysfunction.

## Figures and Tables

**Table 1:** Demographic and clinical characteristics of the whole cohort of MS patients and MS groups based on their CI level.

	MS Cohort (=36)	ACN-MS (=11)	CI-MS (=25)	*p*-Value
Age (years)	46.1 ± 8.1	41.9 ± 8.7	48.0 ± 7.2	*p* = 0.04 *
Education (years)	13.0 ± 3.5	13.9 ± 3.8	12.6 ± 3.3	*p* = 0.29
Gender (M/F)	12/24	2/9	10/15	*p* = 0.20
Disease duration (years)	9.1 ± 7.7	8.6 ± 8.3	9.2 ± 7.6	*p* = 0.83
EDSS	2.5 (0–7)	1.5 (0–3.5)	2.5 (0–7)	*p* = 0.05 *

Legend. ACN = apparently cognitive normal; CI = cognitive impaired; EDSS = Expanded Disability Status Scale; MS = multiple sclerosis; PPMS = primary-progressive multiple sclerosis; RRMS = relapsing-remitting multiple sclerosis; SPMS = secondary-progressive multiple sclerosis; * = significant difference. Mean ± SD were provided for continuous variables; Median [range] is provided for EDSS.

**Table 2:** Significant results from ANCOVA analyses on dMRI indices. For each region of interest (ROI), the difference between the adjusted means of the two groups and their standard errors are reported, along with the statistical significance (Bonferroni-corrected). Hedge’s *g* factors are also included as measures of the effect sizes.

Index	ROI	Mean Difference (CI—ACN)	Std. Error	Sig.	Effect Size (Hedge’s *g*)
FA	Cau	0.019	0.006	0.004	1.030
	Put	0.017	0.007	0.008	0.947
GFA	Cau	0.027	0.014	0.050	0.723
	Put	0.031	0.014	0.026	0.807
	PC	−0.027	0.014	0.045	−0.724
	Pre	−0.030	0.014	0.030	−0.804
PA	Thal	0.022	0.006	0.001	1.047
	Cau	0.020	0.006	0.002	1.039
	Put	379.60	173.941	0.030	0.775
	PCC	−423.12	173.941	0.015	−0.865
RTAP [mm^−2^]	Ins	−369.61	173.941	0.034	−0.754
	LgG	−390.49	173.941	0.025	−0.797
	PC	−378.01	169.358	0.026	−0.773
	Pre	−445.32	169.358	0.009	−0.910
	Put	40,367.72	17,133.29	0.019	0.835
	PCC	−39,119.08	17,133.29	0.023	−0.809
RTOP [mm^−3^]	LgG	−36,666.03	17,133.29	0.033	−0.748
	PC	−32,923.28	16,681.81	0.049	−0.681
	Pre	−41,099.81	16,681.81	0.014	−0.851

Hedge’s g factors were calculated using the pooled standard deviation plus a bias correction for small sample sizes: g = $\frac{M1-M2}{SD_{pooled}^{*}}\cdot \left(1-\frac{3}{4\left(n1+n2\right)-9}\right)$, where *M*_1_ and *M*_2_ are the two group means, *n*_1_ and *n*_2_ are the samples for each group, SD is the standard deviation with $SD_{pooled}^{*}=\sqrt{\frac{\left(n1-1\right)SD_{1}^{2}-\left(n2-1\right)SD_{2}^{2}}{\left(n1+n2-2\right)}}$. Legend. FA = fractional anisotropy; GFA = generalized fractional anisotropy; PA = propagator anisotropy; RTAP = return to the axis probability; RTOP = return to the origin probability; Cau = caudate; Put = putamen; PC = pericalcarine; Pre = precuneus; Thal = thalamus; PCC = posterior cingulate cortex; Ins = insula; LgG = lingual gyrus.

## Data Availability

The data presented in this study are available on request from the senior corresponding author (M.C.). The data are not publicly available due to privacy reasons.

## Supplementary Materials

The following are available online at https://www.mdpi.com/article/10.3390/diagnostics11061103/s1, Supplementary Table S1: Results of ANCOVA analyses comparing the neuropsychological performance of apparently cognitively normal (ACN) and cognitively impaired (CI) patients. Values are reported as mean ± standard deviation across each group.
