# Supplementary material for: Microstructural MRI Correlates of Cognitive Impairment in Multiple Sclerosis: The Role of Deep Gray Matter
Source: Diagnostics (Basel). 2021 Jun 16;11(6):1103. doi: 10.3390/diagnostics11061103 (PMC8234586; doi:10.3390/diagnostics11061103)
Supplement: Supplementary file 1 [file diagnostics-11-01103-s001.zip › diagnostics-1195867-supplementary.pdf]

Supplemental Material

Table S1. Results of ANCOVA analyses comparing the neuropsychological performance of apparently cognitively normal (ACN) and cognitive impaired (CI) patients. Values are reported as mean ± standard deviation across each group.

Test	CI	ACN	Sig.
**SRT_LTS**	29.92 ± 2.70	50.98 ± 4.19	<0.001*
**SRT_CLRT**	20.19 ± 2.68	43.56 ± 4.19	<0.001*
**SRT_D**	5.64 ± 0.49	9.27 ± 0.76	0.001*
**SPART_I**	19.18 ± 0.87	23.95 ± 1.35	0.007*
**SPART_D**	6.67 ± 0.39	8.75 ± 0.61	0.009*
**SDMT**	43.52 ± 2.16	56.71 ± 3.35	0.003*
**PASAT3**	36.65 ± 1.99	47.69 ± 3.09	0.007*
**PASAT2**	29.14 ± 1.98	36.65 ± 3.08	0.055
**WLG**	22.94 ± 0.99	29.04 ± 1.54	0.003*
**ST_EIT**	21.97 ± 4.72	11.38 ± 7.33	0.248
**ST_EIE**	1.13 ± 0.33	0.02 ± 0.52	0.091

*SRT_LTS = selective reminding test-long-term storage; SRT_CLRT = selective reminding test-consistent long-term retrieval; SRT_D = selective reminding test-delayed; SPART_I = spatial recall test-immediate; SPART_D = spatial recall test-delayed; SDMT = symbol digit modalities test; PASAT = paced auditory serial addition test; WLG = world list generation; ST_EIT = stroop test-effect interference time; ST_EIE = stroop test-effect interference error; * = statistical significance at p<0.05*
